# Radiation- and Radical-Induced Graft Copolymers for Environmental Remediation and Separation Technologies

**DOI:** 10.3390/ma19163499

**Published:** 2026-08-18

**Authors:** Nelson Rotich Kiprono, Stephen Kabasa, Geeva Prasanth Annamalaisamy, Hanna Lewandowska

**Affiliations:** 1 Institute of Nuclear Chemistry and Technology, Dorodna 16, 03-195 Warsaw, Poland; n.kiprono@ichtj.waw.pl (N.R.K.); s.kabasa@ichtj.waw.pl (S.K.); g.prasanth@ichtj.waw.pl (G.P.A.); 2 School of Health & Medical Sciences, Vizja University, Okopowa 59, 01-043 Warsaw, Poland

**Keywords:** graft copolymerization, radiation-induced grafting, environmental remediation, separation membranes, radionuclide recovery, water purification

## Abstract

**Highlights:**

- Radiation-induced grafting enables initiator-free functionalization of polymer surfaces with the potential to preserve bulk properties when dose, substrate, and atmosphere are appropriately controlled.
- Controlled radical approaches, especially RAFT, improve control over graft density, chain length, and architecture.
- Radiation- and radical-grafted membranes, sorbents, and composites offer tunable platforms for separations, water treatment, and radionuclide remediation.

**Abstract:**

Modern separation and purification technologies increasingly require materials that combine high selectivity, chemical robustness, and long-term operational stability without compromising mechanical performance. Radiation- and radical-induced graft copolymerization addresses this need by generating radical sites on polymer backbones and introducing tailored functional groups through subsequent monomer grafting. Retention of bulk properties, however, depends on controlling radiation dose, polymer structure, oxygen, and irradiation conditions so that grafting is favored over chain scission, crosslinking, and embrittlement. This review critically examines recent grafting strategies for gas and liquid separation, water treatment, radionuclide management, and resource recovery. It relates radical generation, graft growth, structural control, and functional-group chemistry to material performance and process optimization. Attention is given to radiation-induced grafting and its integration with controlled radical polymerization, especially reversible addition–fragmentation chain-transfer polymerization, to regulate graft density, chain length, and architecture. Composite and interfacial approaches are also evaluated. The review discusses the requirements and remaining barriers to practical translation, including dose optimization, long-term stability, regeneration, reproducibility, scalability, and the need for techno-economic and life-cycle assessments.

## 1. Introduction

Radiation-induced grafting can reduce the need for chemical initiators, thereby limiting initiator-derived by-products and contamination of the modified material. Because radicals are generated in situ, the method is not constrained by initiator diffusion and can support localized modification at phase boundaries or internal surfaces. However, preservation of bulk properties depends on controlling dose, dose rate, oxygen, temperature, substrate crystallinity, and irradiation environment; otherwise chain scission, crosslinking, oxidation, or embrittlement may become significant. The process can also be carried out under sterile conditions, which is valuable for biomedical applications [1,2,3,4].

Polymeric selective materials, comprising membranes with unique features, have expanded rapidly over the last several decades, thanks to enhanced methods used to develop structures suited for applications in diverse separation processes. These newly established functional materials come in multiple physical forms, namely membranes, hydrogels, adsorbents, and resins that cover an extensive range of separation purposes. Functional copolymers formed by methods such as Radiation-Induced Graft Copolymerization (RIGC) of nonpolar or polar monomers onto conventional polymers offer an appealing type of separation surface. It results in improved characteristics caused by the addition of hydrophilic ionic groups that enormously reconfigure the backbone polymers [1,2,5].

Previous reviews have generally treated radiation grafting, surface modification of separation membranes, controlled radical polymerization, and specific applications such as uranium recovery as separate topics [1,4,6,7,8,9]. The distinctive contribution of this narrative review is to connect these strands within one application-focused framework: it compares conventional free-radical grafting with ATRP, NMP, RAFT, and radiation-induced grafting; links graft architecture and interfacial chemistry to separation performance; and evaluates membranes, composite sorbents, and radionuclide-recovery systems together. Throughout this article, “radiation-induced grafting” (RIG) is used as the general term, whereas “radiation-induced graft copolymerization” (RIGC) is reserved for cases in which graft-chain polymerization is specifically discussed.

### 1.1. Methodology and Scope of the Narrative Review

This article is a narrative review rather than a systematic review or meta-analysis. The literature search was conducted in Google Scholar and was last updated in July 2026. Searches were performed using combinations of terms related to radiation-induced grafting, radical graft copolymerization, controlled radical polymerization, membranes, adsorption, environmental remediation, gas and liquid separation, water treatment, radionuclide removal, and resource recovery. Reference lists of relevant review articles and primary studies were additionally examined to identify further publications. Priority was given to peer-reviewed publications from the preceding ten years, while older studies were retained when they established mechanisms, methods, or historically important applications. Eligible studies addressed radical- or radiation-induced grafting of polymeric substrates and reported mechanistic, materials, separation, adsorption, or stability information relevant to environmental remediation, gas or liquid separation, water treatment, radionuclide management, or resource recovery. Studies outside this scope, non-polymeric systems without a grafting component, and reports lacking sufficient methodological or performance detail were not used as core evidence. Evidence was synthesized according to grafting mechanism, substrate and functional group, target separation, reported performance, operating conditions, stability, and implementation limitations.

AI-assisted tools were used only as supporting graphical tools during the preparation and aesthetic enhancement of selected visual elements in figures. This included generating or refining individual graphical components, schematic objects, simplified structural illustrations, and layout-supporting graphical elements based on author-defined instructions or source materials. No complete figure was generated autonomously by AI. The scientific concept, final arrangement, labels, captions, descriptions, and interpretation of all figures were prepared, edited, and verified by the authors.

Generative artificial intelligence tools were not used to generate scientific conclusions, interpret data, select literature, or write the scientific content of the review.

### 1.2. Fundamentals of Graft Polymerization and Radiation-Induced Grafting

Grafting involves the attachment of one polymer, called the “graft,” onto another polymer matrix, referred to as the “backbone”. By grafting, it is possible to modify or enhance the properties of the base polymer, thus introducing new characteristics and functionalities. Grafting can be used to modify solubility [10], nano-dimensional morphology [11], biocompatibility [12], and bio-responsiveness [13]. It can also be utilized to enhance charge transport properties [14,15]. Among the various chemical approaches for polymer grafting are free radical reactions, click reactions, amide formation, and alkylation. While these methods offer diverse routes to polymer functionalization, this review focuses specifically on radical- and radiation-induced grafting techniques.

**Figure 1:**
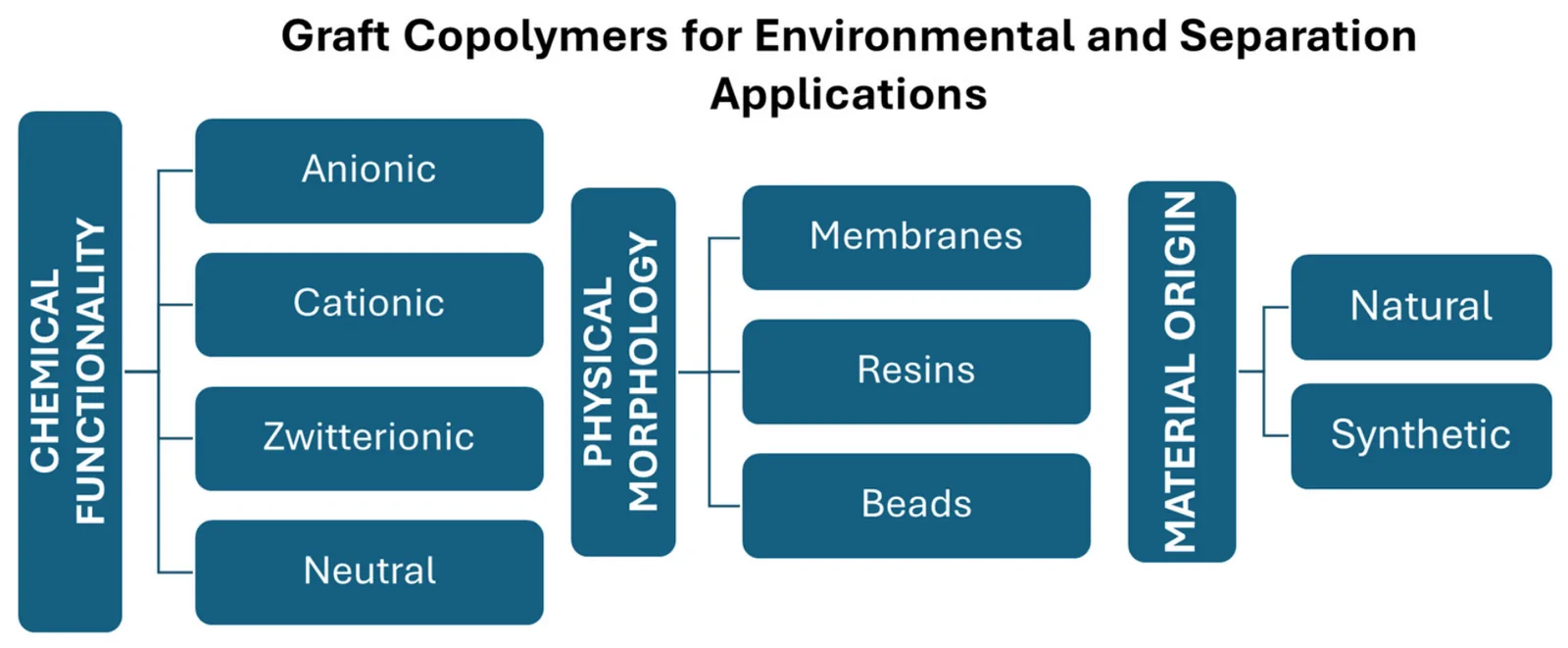
Faceted classification of radical- and radiation-induced graft copolymers for environmental remediation and separation. Author-created synthesis based on [1].

Grafted surfaces can be neutral or ionic, depending on the nature of the grafted monomer and the accompanying chemical processes for imparting the ionic properties. They can be categorized into several broad classes based on their function or mechanism of separation, which include ion exchangers, graft copolymers, synthetic graft adsorbents, grafted bio-adsorbents, and chelating copolymers, amongst others, as shown in Figure 1 [1].

### 1.3. Methods of Graft Copolymerization

Several approaches have been developed to achieve graft copolymerization. Conventional free-radical grafting relies on thermal or chemical initiators and offers broad tolerance to substrates and solvents, but generally provides limited control over chain architecture and molecular weight [16,17]. Classical living polymerizations, based on anionic or cationic mechanisms, suppress irreversible termination and chain transfer; growing chains therefore remain active, which supports narrow molecular-weight distributions and complex architectures [17,18,19]. Nonetheless, they demand highly purified reagents, strictly anhydrous conditions, and often low polymerization temperatures. Classical anionic and cationic polymerization methods are constrained to a limited range of monomers due to their sensitivity to common functional groups found in vinyl monomers, such as halogens, nucleophilic functional groups, and acidic protons [20,21]. A more versatile alternative is controlled radical polymerization (CRP), which enables controlled chain growth in radical systems through principles analogous to those governing classical living polymerization. The concept of living polymerization was originally established in anionic and cationic systems [18]. Living polymerization is achieved by ensuring that the active chain ends remain protected against termination and chain transfer. All chains are initiated rapidly at the beginning of the reaction, and no significant initiation occurs thereafter. The propagating centers can be carbanions in anionic systems, carbocations in cationic systems, or radicals in controlled radical polymerizations. In classical ionic systems, this protection arises from the inherent absence of side reactions under rigorously controlled conditions, so that all chains initiated at the start remain active until monomer is consumed. In modern controlled radical polymerizations, the same principle is implemented through a reversible activation–deactivation equilibrium: the propagating radical is repeatedly converted into a dormant species by a mediating agent. Here, CRP encompasses several mechanistically distinct strategies, including atom transfer radical polymerization (ATRP), in which reversible activation is mediated by transition-metal complexes [22,23], nitroxide-mediated polymerization (NMP), which relies on reversible capping of growing chains by nitroxide radicals [24,25,26,27,28], and reversible addition–fragmentation chain transfer (RAFT) polymerization, which controls chain growth through reversible chain transfer using thiocarbonylthio compounds [16,29,30,31,32,33]. Detailed information on various living polymerization methods can be found in [17,19].

**Figure 2:**
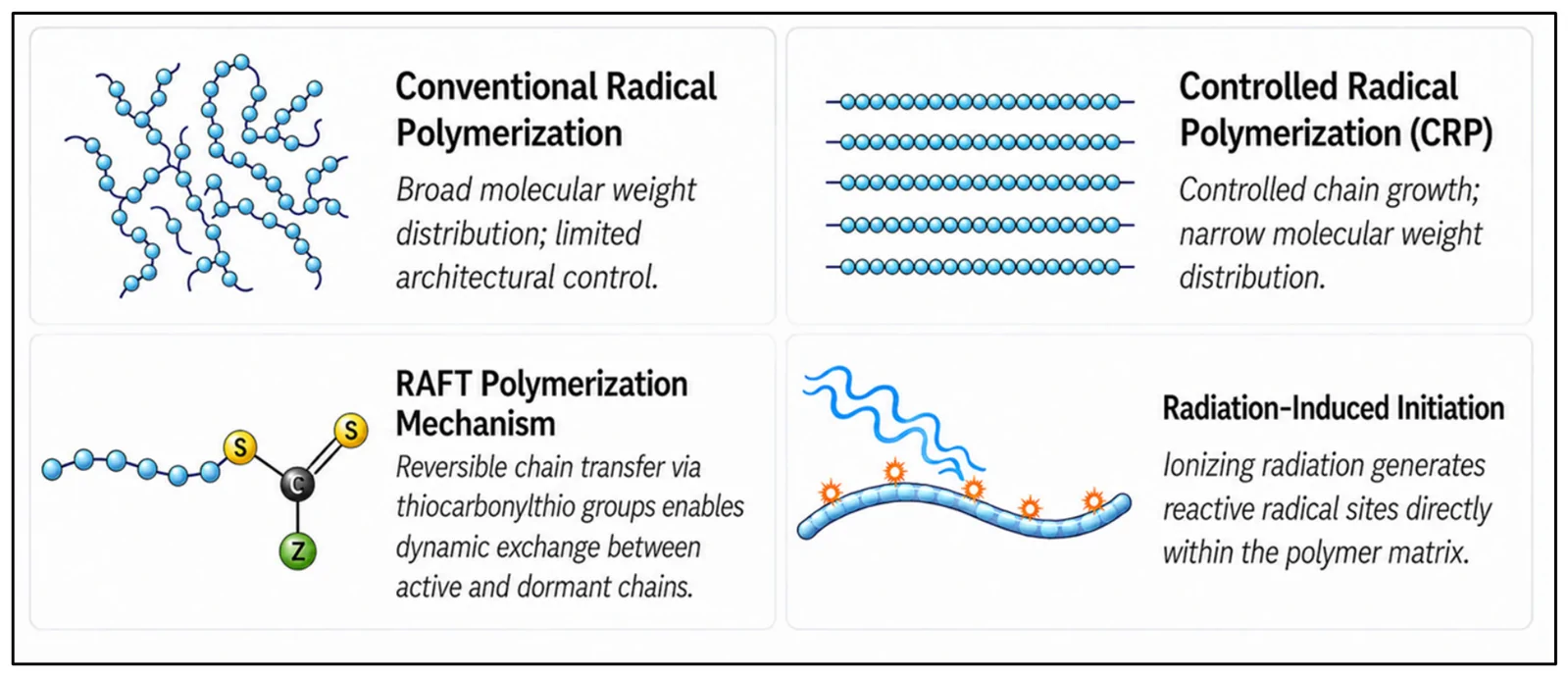
Schematic comparison of conventional radical polymerization, controlled radical polymerization (CRP), RAFT-mediated control, and radiation-induced initiation. Conventional radical polymerization generally provides limited architectural control and broad molecular-weight distributions, whereas CRP regulates chain growth through reversible activation, deactivation, or transfer processes. RAFT is shown as a representative CRP mechanism based on reversible thiocarbonylthio-mediated chain transfer. Ionizing radiation provides a distinct means of generating radical sites directly within polymer substrates. Polymer repeat units are shown in blue. In the RAFT group, Z denotes the substituent that controls its reactivity. Orange asterisks mark radiation-generated radical sites.

Among these methods, RAFT is particularly noteworthy as it allows the controlled polymerization of a broad range of functional monomers under mild reaction conditions, including tolerance to oxygen and low temperatures [29]. The principal distinctions among conventional radical polymerization, controlled radical polymerization, RAFT-mediated control, and radiation-induced initiation are summarized schematically in Figure 2 and Table 1.

### 1.4. Reversible Addition–Fragmentation Chain Transfer (RAFT) Polymerization

**Figure 3:**
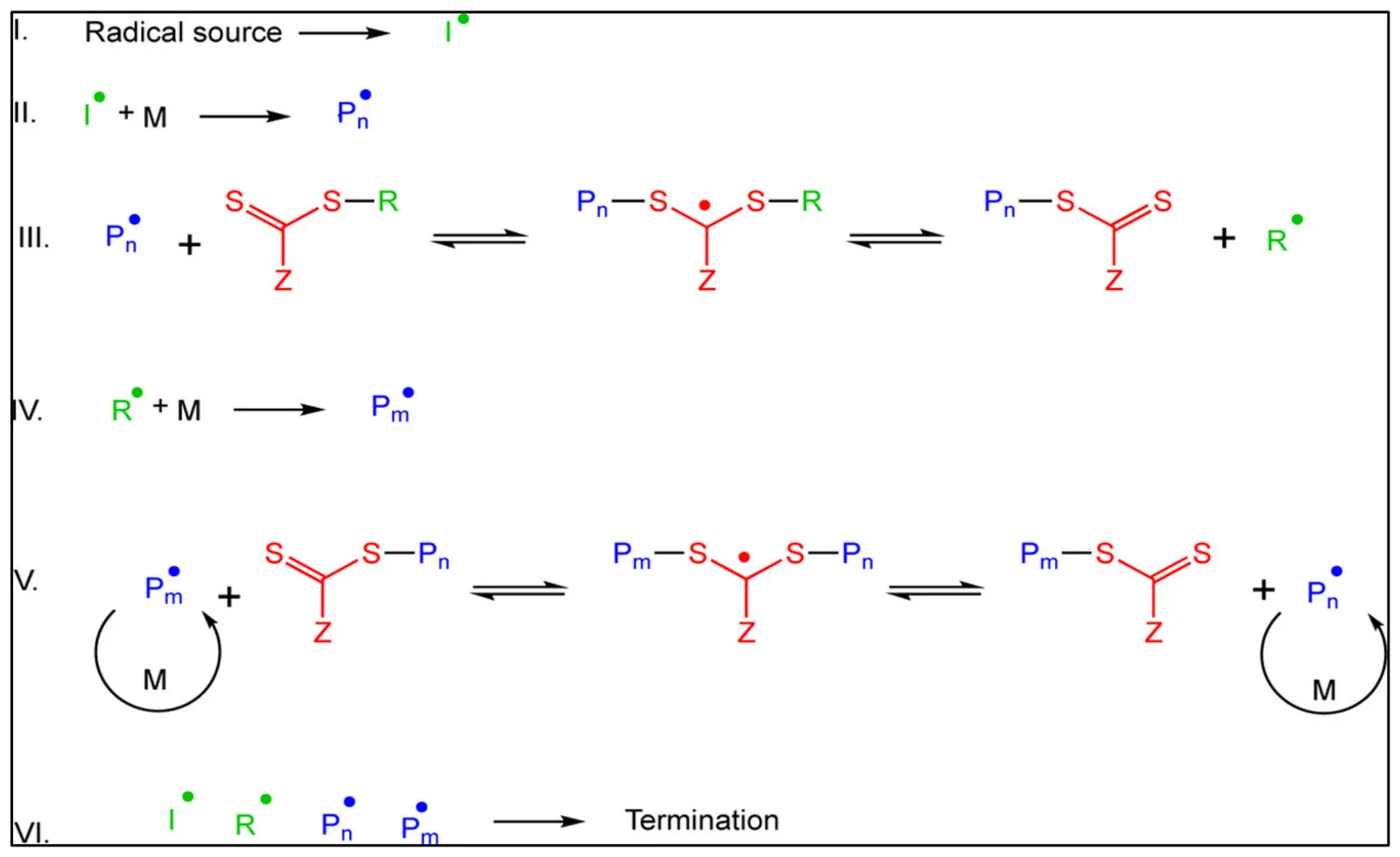
RAFT polymerization mechanism. (I) radical generation; (II) initiation and chain propagation; (III) RAFT pre-equilibrium through radical addition and fragmentation; (IV) reinitiation by the leaving-group radical; (V) reversible exchange between propagating radicals and dormant thiocarbonylthio-capped chains; and (VI) radical termination. Reprinted from [35] with the permission of the American Chemical Society.

RAFT achieves living character through the use of a thiocarbonylthio chain transfer agent (CTA, Figure 3), which mediates a rapid and reversible exchange between active propagating radicals and dormant species. The use of a CTA as a chain transfer agent in living free-radical polymerization was proposed by Chiefari et al. in 1998 [29,34]. In RAFT, the effectiveness of thiocarbonylthio-based CTAs comes from the kinetic balance between the radical addition rate to the C=S bond and the β-fragmentation rate of the adduct radical. For common CTA–monomer systems, these values are typically on the order of 10^3^–10^6^ M^−1^·s^−1^ for addition and 10^5^–10^7^ s^−1^ for fragmentation, depending on monomer type, CTA substituents, and temperature. This CTA system enables the synthesis of polymers with narrow polydispersities and a linear increase of molecular weight with conversion, while preserving active end groups that can be re-initiated for further chain growth or block copolymer formation [29,30].

Figure 3 illustrates the RAFT mechanism, which involves activation (step I) followed by equilibrium between active and dormant species (steps III and V) through the addition of radicals to the RAFT agent (CTA). The key chain transfer steps in the RAFT mechanism involve the reversible transfer of the functional chain end-group (often a thiocarbonylthio group, Z–C(=S)S–R) between dormant chains (macroRAFT agent or macroCTA) and propagating radicals. The insertion of monomers between the R- and Z–C(=S)S-groups of a RAFT agent forms the α and ω end-groups of the resulting polymeric chains, ensuring that all chains have a similar degree of polymerization (DP) at any given time [35,36,37].

Radiation-induced grafting has been used for decades to modify polymer surfaces and internal pore walls [3]. Exposure to ionizing radiation forms radicals on or within the substrate, which initiate graft-chain growth after contact with the selected monomer. Dose, dose rate, irradiation atmosphere, temperature, and exposure time must be optimized so that grafting competes successfully with termination, homopolymerization, crosslinking, oxidative degradation, and chain scission. The non-selective radical initiation enables attachment of polar or non-polar monomers and introduction of groups such as –COOH, –OR, –OH, –NH_2_, and –SO_3_H. Modification may remain surface-confined or extend into the bulk depending on radiation penetration and monomer diffusion. Consequently, bulk properties can be retained only within an appropriate process window and for a compatible substrate; polymer type, crystallinity, oxygen, and irradiation environment are decisive [3,4,38,39,40]. Films, membranes, fibres, and complex geometries can all be treated, but radiation-sensitive matrices require lower doses, protective conditions, or alternative initiation strategies.

**Figure 4:**
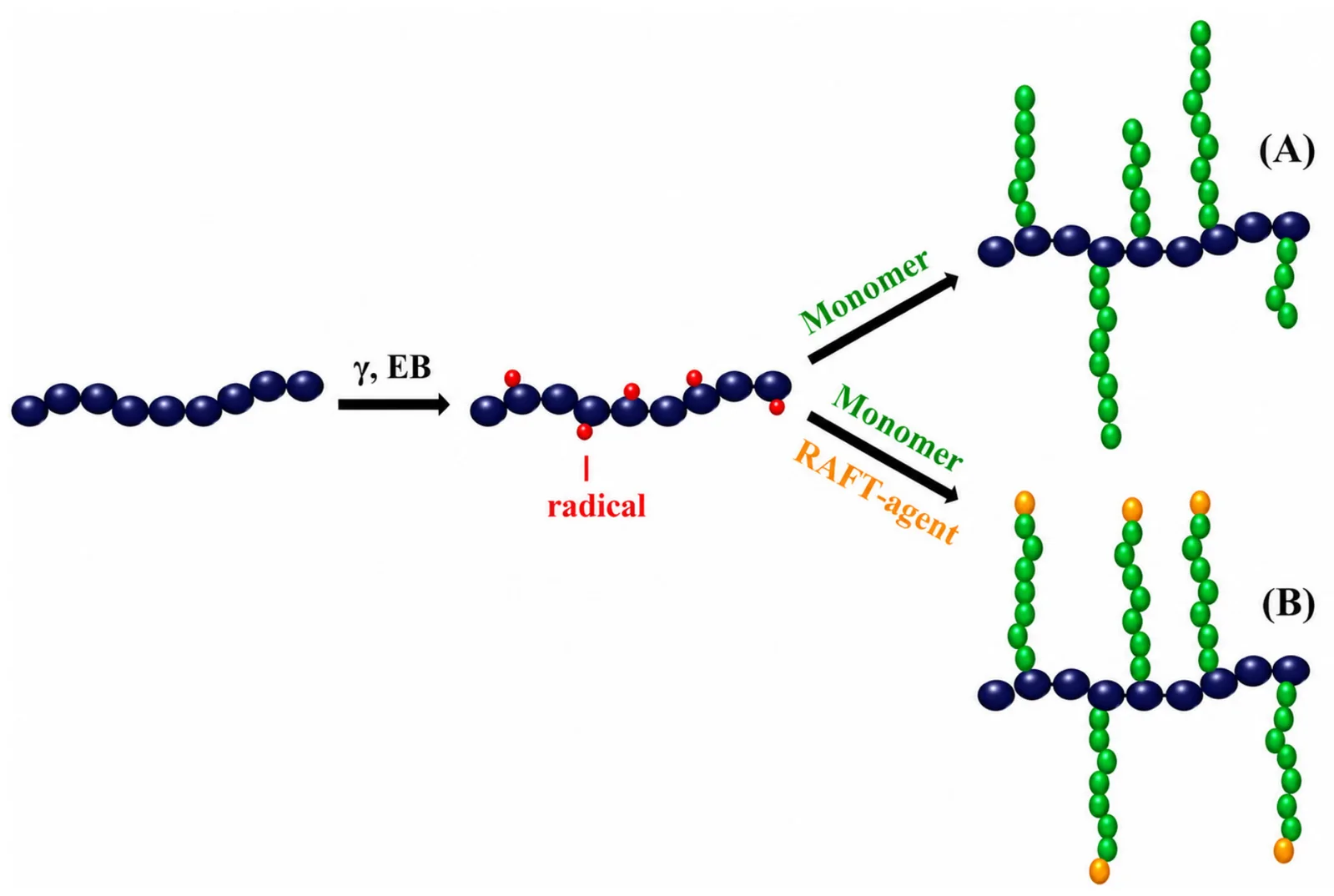
Radiation-induced grafting using (**A**) conventional and (**B**) RAFT-mediated polymerization initiated by ionizing radiation. Dark blue spheres denote the polymer backbone; green spheres, grafted repeat units; red markers, radical sites; yellow spheres, RAFT-derived thiocarbonylthio end groups. Enhanced, from [46] with permission of the Institute of Nuclear Chemistry and Technology.

Radiation can serve as an initiation method in RAFT polymerization, providing an alternative to conventional thermal or chemical initiators. Several radiation-based techniques can be used for this purpose, including photo-initiators, electron-beam irradiation, microwave irradiation, and gamma irradiation (Figure 4). These radiation-induced methods offer alternative options for initiating RAFT polymerization reactions, providing versatility and expanded possibilities in polymer synthesis [41,42,43]. Among these methods, gamma-irradiation and ultraviolet (UV) radiation are advantageous, particularly for bioconjugate chemistry. This is because they allow initiation at low temperatures to prevent protein/biomolecule denaturation [44]. Gamma irradiation has the additional benefit of being more penetrating and non-selective, making it suitable for commercial-scale production. Furthermore, gamma-initiated living free-radical polymerization is environmentally friendly and does not require catalysts or initiators, operating at room temperature. Additionally, gamma-irradiation is an excellent method for generating radicals on both organic and inorganic substances, making it highly valuable for the controlled synthesis of graft copolymers [41,42,43,45]. An additional advantage of radiation-induced initiation lies in the ability to control the spatial distribution of radical formation by selecting radiation with different penetration depths. Low-penetrating radiation, such as ultraviolet (UV) light, is primarily absorbed at or near the material surface, making it particularly suitable for surface modification and the formation of grafted layers. In contrast, highly penetrating radiation, such as gamma irradiation, enables radical generation throughout the bulk of the material. This tunability allows precise control over whether polymerization is confined to the surface or occurs uniformly within the entire material, depending on the intended application [4].

The combination of living polymerization and radical grafting can be a powerful approach to creating complex polymer architectures with controlled grafting densities and precise molecular weights [47]. Such approaches allow for precise tailoring of polymer properties, improving their functionality in specific applications. Moreover, the versatility of graft polymers makes them ideal candidates for developing advanced materials with unique properties. In this context, the present review highlights how the integration of radiation-induced grafting and controlled radical polymerization is reshaping the design of functional polymer systems. The subsequent sections focus on their application in separation technologies, including gas and liquid membranes, water treatment systems, and radionuclide recovery platforms. Attention is given to how molecular-level control, interfacial engineering, and composite strategies enable the development of next-generation materials with enhanced selectivity, stability, and scalability.

## 2. Graft Polymers in the Separation of Gases and Liquids

Separation processes account for a large share of industrial resource use: in many chemical and refining operations they represent roughly 40–90% of total capital and operating costs. Thermally driven separations such as distillation consume about 10–15% of global energy use, whereas membrane separations can operate at approximately 0.1–2 kWh m^−3^, depending on the feed and process configuration [6,48]. This energy advantage has driven the development of membranes, adsorption media, and hybrid processes that aim to increase selectivity and productivity while reducing solvent use, emissions, and process intensity.

In recent years, membranes, hydrogels, and resins have become increasingly important in separation technologies [1]. Grafting can introduce targeted surface functionality while preserving the principal bulk properties of the substrate [6]. Polymer-functionalized nanostructures may also enhance analytical performance through mechanisms such as optical scattering, which can amplify signals in sensing applications [49].

Poly(vinyl chloride) (PVC) is widely used because of its low cost and resistance to acids and bases [50]. Incorporating polar, water-soluble monomers into PVC by conventional copolymerization is difficult because the components partition differently between aqueous and organic phases [51]. Fang et al. [51] therefore prepared PVC-g-poly(dimethylaminoethyl methacrylate) (PVC-g-PDMA) by ATRP, using labile chlorine atoms on the PVC backbone as initiation sites [51,52]. The graft copolymer was processed into a nanofiltration membrane by nonsolvent-induced phase separation. PDMA grafts increased hydrophilicity and permeability, while partial quaternization generated a positively charged surface that rejected cationic Victoria blue B through Donnan exclusion. The reported dye rejection was approximately 91.2%. The hydrophilic, cationic grafts also reduced foulant interactions and provided antibacterial activity, although the comparatively thick selective layer remains a practical limitation [51].

Chitosan and other polysaccharide-based materials have attracted increasing interest for membrane applications due to their biodegradability, inherent hydrophilicity, abundant functional groups, and favorable mechanical properties. These characteristics make chitosan a promising candidate for separation membranes, particularly in aqueous and environmentally oriented applications. However, the practical modification of polysaccharides remains challenging because their sugar backbones are highly sensitive to harsh chemical conditions. Conventional radiation-induced grafting methods are generally unsuitable for polysaccharides, as high-energy irradiation can cause backbone scission and degradation, leading to loss of mechanical integrity and performance. Consequently, alternative grafting strategies that operate under milder conditions are required to functionalize chitosan without compromising its structure [53].

Conventional nano- and subnanoporous membranes are frequently prepared by interfacial polymerization of m-phenylenediamine and trimesoyl chloride, a process that may require substantial quantities of alkane solvents. This increases cost and creates additional environmental concerns [54]. Blended nanofiltration membranes provide one alternative route [55], whereas covalent grafting offers a distinct strategy for introducing persistent functionality into or onto a membrane substrate.

Liu et al. [56] grafted catechin, a natural water-soluble polyphenol, onto chitosan and deposited the resulting conjugate on a hydrolyzed polyacrylonitrile ultrafiltration membrane. Hydroxyl radicals (•OH), generated by the reaction of vitamin C with H_2_O_2_ [57], abstracted hydrogen from chitosan amino and hydroxyl groups to form chitosan macroradicals. These reactive sites subsequently coupled with catechin [58]. The membrane rejected 99.6% of Congo red, 98.7% of acid fuchsin, and 98.5% of crystal violet while retaining comparatively little inorganic salt and showing favorable dye-antifouling behavior. The essential role of grafting in this system is to produce chemically modified chitosan containing covalently attached, persistent polyphenolic functionality. Its principal process limitation is competing catechin self-polymerization, which can generate poly(catechin) deposits and therefore requires careful control of the radical reaction [56].

Composite formation offers a complementary strategy for modifying membranes used in aqueous dye separation. Brahmi et al. [59] incorporated 3 wt% polyaniline (PANi) into a polysulfone (PSF) casting solution and prepared PSF/PANi membranes by phase inversion. Adding PANi reduced the water-contact angle from 85.12° to 39.45°, increased porosity from 63.12% to 86.08%, and increased equilibrium water content from 36% to 83%. Under optimized conditions for a binary dye mixture, the membrane rejected approximately 96% of methylene blue and 91% of Congo red. These effects were attributed to changes in hydrophilicity and pore structure, together with hydrogen bonding, π–π interactions, electrostatic interactions, and charge-transfer effects between the membrane and the dyes. Unlike catechin grafting, this approach introduces a pre-existing functional polymer without covalent attachment to the PSF backbone and can modify membrane composition, morphology, and dye affinity in a single fabrication step. Although PANi leaching was not demonstrated, long-term PANi retention should be assessed alongside permeate flux, fouling, regeneration, mechanical stability, and performance in realistic wastewater [59].

Beyond dye separation, chitosan-based graft copolymers have also shown promise in gas separation. Otvagina et al. [60] prepared chitosan-g-polystyrene and chitosan-g-polyacrylonitrile membranes containing methylimidazolium-based ionic liquids for CO_2_ separation. Grafting synthetic polymer chains and incorporating ionic liquids reduced chitosan crystallinity and enhanced gas permeability without sacrificing selectivity [61]. Ionic liquids containing [BF_4_]^−^, [PF_6_]^−^, and [Tf_2_N]^−^ anions were selected because of their high CO_2_ solubility [62,63]. The graft copolymers also retained high tensile strength, approximately 75–104 MPa for the chitosan-g-polyacrylonitrile system and 69–75 MPa for the chitosan-g-polystyrene system [60].

Despite these advances, grafting polysaccharides remains intrinsically challenging because their backbones may undergo degradation during both chemically and radiation-initiated radical reactions. Chitosan has been grafted with vinyl monomers using initiators such as AIBN, persulfates, and redox systems [64,65], but peroxide-based and redox systems can promote backbone degradation and homopolymer formation [66]. Alternative initiation systems based on Ce(IV) or Co(III) complexes may provide higher grafting efficiency under less aggressive conditions. Baranov et al. [66] used hexaamminecobalt(III) chloride to graft acrylonitrile and methyl acrylate onto chitosan, achieving efficient grafting without detectable degradation of the polysaccharide backbone [66].

In industrial CO_2_ separation, chemical absorption in aqueous amine solutions remains an established technology. CO_2_ is absorbed through reversible reaction with amines, after which the solvent must be heated for regeneration; this step imposes substantial energy and operating costs [67]. Polymeric membrane separation offers simpler operation and can reduce energy consumption relative to solvent regeneration [68], but polymer membranes are constrained by the permeability–selectivity trade-off. Mixed-matrix membranes address this limitation by incorporating inorganic fillers into polymer matrices.

Poly(oxyethylene methacrylate) (POEM) is attractive for CO_2_ separation because its oxyethylene groups provide ether-oxygen sites with affinity for CO_2_ [69]. Kim et al. [70] prepared a P25 TiO_2_/PVC-g-POEM mixed-matrix membrane in which the PVC-g-POEM graft copolymer was synthesized by ATRP. PVC-g-POEM alone exhibited a CO_2_ permeability of 46.2 Barrer and a CO_2_/N_2_ selectivity of 29.6. Incorporation of P25 increased permeability by 122% to 102.8 Barrer and increased CO_2_/N_2_ selectivity by 32% to 39.1 [70].

Olefin/paraffin separation can be facilitated by reversible complexation between olefins and Ag^+^ carriers in liquid membranes [71]. Hsiue et al. [72] prepared polyethylene, poly(1-trimethylsilyl-1-propyne), and silicone-rubber membranes by pre-irradiation grafting of acrylic acid followed by Ag^+^ complexation. Gamma pre-irradiation in air generated peroxides and hydroperoxides, after which acrylic acid was grafted under nitrogen in the presence of Mohr’s salt [73]. Increasing the degree of acrylic-acid grafting reduced permeability. PE-g-AA-Ag^+^ showed low permeability but high olefin/paraffin separation, whereas SR-g-AA-Ag^+^ and PTMSP-g-AA-Ag^+^ showed higher permeability with low-to-moderate separation performance [72].

Pervaporation with PDMS-containing graft copolymers provides a non-radiation route for separating volatile organic compounds from water. In pervaporation, a liquid feed contacts one side of a selective membrane and permeating components are removed as vapor under reduced downstream partial pressure [74]. Akimoto et al. [75] prepared PDMS-g-poly(amide-imide) membranes and observed preferential permeation of ethanol, acetone, and tetrahydrofuran [75]. Nagase et al. [76] synthesized polyimide-g-PDMS by polycondensation followed by thermal treatment. The graft copolymer was insoluble in the tested organic solvents, and increasing PDMS content enhanced gas permeability. Membranes containing more than 50 wt% PDMS exhibited oxygen permeability above 10^−8^ cm^3^(STP) cm^−2^ s^−1^ cmHg^−1^ and preferentially transported ethanol, acetonitrile, and acetone from aqueous mixtures over a broad feed-composition range at 50 °C [76].

Recent advances in radiation-induced grafting have demonstrated the feasibility of precise structural re-engineering of microporous membranes for high-performance gas separation. A notable example is the development of polyolefin reweaved ultra-micropore membranes (PRUMs), in which electron beam (EB) irradiation induces in situ polymerization within pre-existing porous scaffolds [77].

PIM-1, a representative polymer of intrinsic microporosity (PIM), and Tröger’s base (TB) polymers are two closely related classes of high-performance microporous materials commonly employed as such scaffolds. PIM-1 exhibits a rigid ladder-type structure with a highly contorted backbone, resulting in exceptionally high fractional free volume and gas permeability, whereas TB-based polymers form shape-persistent frameworks with comparable intrinsic microporosity. Chemically, PIM-1 typically contains heteroatoms such as oxygen, while TB polymers can be composed solely of carbon and nitrogen, enabling selective tracking of oxygen-containing grafted species within the matrix. Despite their high permeability, both PIM-1 and TB-based polymers suffer from intrinsic limitations, including physical aging, plasticization under high gas pressures, and limited long-term stability, which restrict their practical application in industrial gas separation.

**Figure 5:**
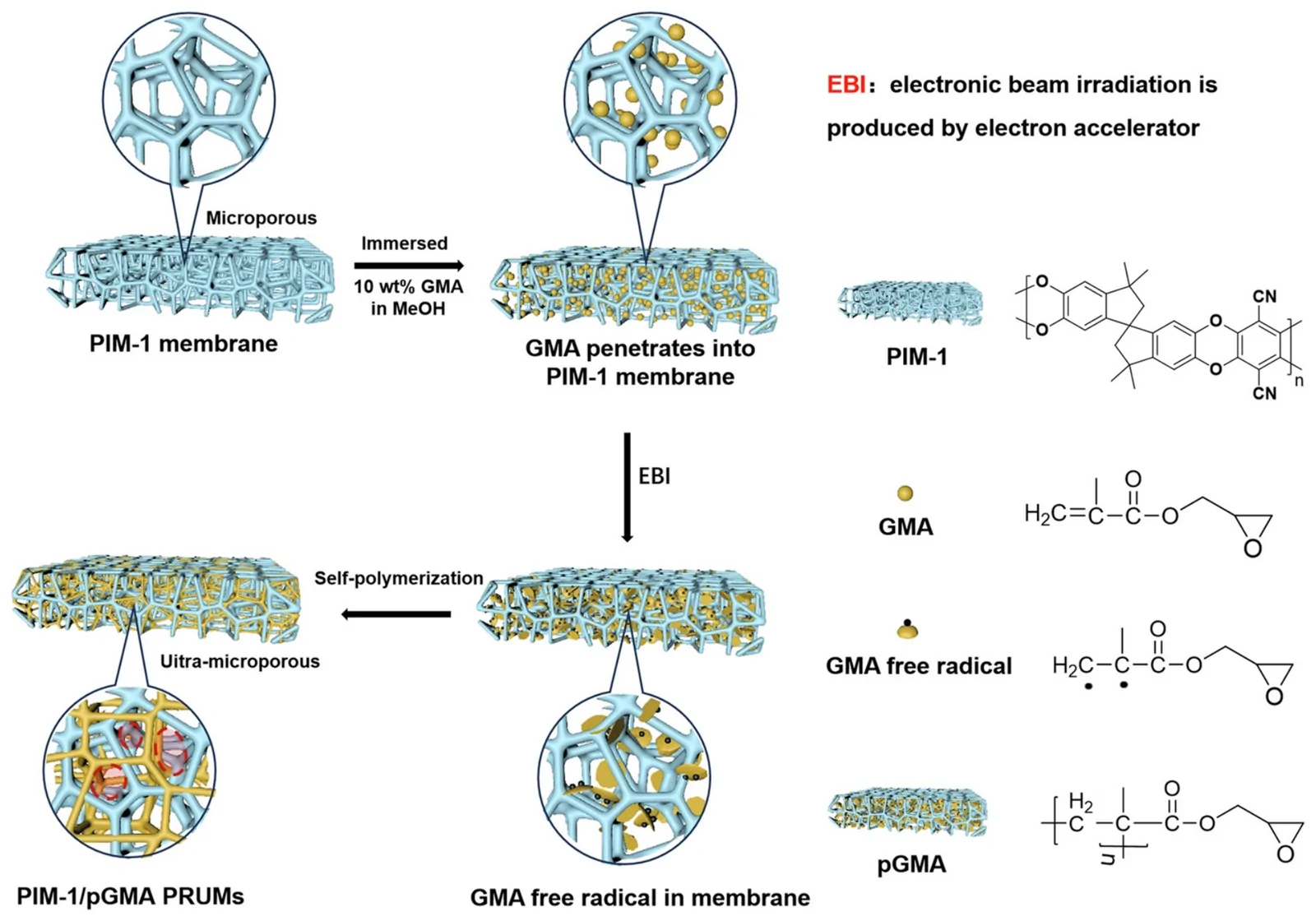
Electron-beam-induced in situ polymerization of glycidyl methacrylate (GMA) within a PIM-1 membrane and formation of PIM-1/pGMA polyolefin reweaved ultra-micropore membranes (PRUMs). Reprinted from [77]. © The Author(s) 2025. The original figure is licensed under CC BY-NC-ND 4.0 (https://creativecommons.org/licenses/by-nc-nd/4.0/, accessed on 7 August 2026).

In the PRUM approach, pristine membranes (e.g., PIM-1 or TB, 50–60 μm thickness) are infiltrated with a 10 wt% solution of glycidyl methacrylate (GMA) in methanol, and irradiated with a 1 MeV electron beam at doses of 20–80 kGy in air. Subsequent post-treatment in a water bath at 40 °C for 3 h ensures completion of free-radical polymerization, and residual homopolymer is removed by ultrasonic washing in methanol. EB irradiation simultaneously generates radical sites within both the scaffold and monomer phase, inducing formation of rigid poly(GMA) segments that effectively reweave the microporous network. The process is illustrated schematically in Figure 5.

This process produces defect-free ultramicroporous nanochannels with highly controlled size distribution. As a result, CO_2_ permeability reaches 1976 Barrer, exceeding the 2019 Robeson upper bound for both CO_2_/CH_4_ and CO_2_/N_2_ separations thereby overcoming the conventional permeability–selectivity trade-off.

Taken together, the catechin-grafted chitosan, PSF/PANi, and PIM-1/pGMA systems illustrate three complementary modes of membrane functionalization. Covalent grafting chemically anchors introduced functionality to a polymer backbone; composite formation incorporates a pre-existing functional material into the membrane-forming matrix; and in situ polymerization generates a second polymer directly within a pre-existing nanostructure. These systems cannot be directly ranked by rejection or permeability because they address different separation problems, phases, and test conditions. They can, however, be compared in terms of functional-component localization, mode of retention, structural effect, fabrication complexity, and long-term stability. Strategy selection therefore depends not only on initial separation performance but also on resistance to component leaching or structural rearrangement, regeneration behavior, and stability under realistic operating conditions.

The same design principles that govern membrane separation—controlled placement of functional groups, accessible transport pathways, and resistance to swelling or fouling—also govern radionuclide capture. The next section therefore shifts from molecular permeation to selective ion binding, where grafted chains act as chelating, ion-exchange, or ion-imprinted domains on mechanically processable supports.

In summary, across PVC, chitosan, mixed-matrix, pervaporation, and radiation-restructured membranes, grafting improves performance when it changes the interfacial chemistry or transport-pathway dimensions without creating an excessively thick, crosslinked, or unstable selective layer. Reported permeability, rejection, and adsorption values are not directly interchangeable because feed composition, pressure, temperature, membrane thickness, ageing, and fouling protocols differ. Industrially relevant comparisons therefore require matched operating conditions, cyclic tests, and reporting of both productivity and selectivity over time.

## 3. Separation of Radionuclides Using Grafted Polymer Sorbents

The growing use of nuclear energy and medical radionuclides has increased the generation of radioactive waste containing species such as ^60^Co^2+^, ^90^Sr^2+^, ^137^Cs^+^, and ^152^Eu^3+^ [78,79]. Their persistence and, in many cases, penetrating γ radiation create environmental and health risks. At the same time, radioisotope production requires selective separations that provide high radiochemical and radionuclidic purity. Grafted polymers are therefore being investigated both for waste remediation and for radioisotope recovery because they can combine reusable formats with high sorption capacity [78]. Representative targets include cobalt, strontium, cesium, europium, uranium(VI), scandium(III), technetium-99m, and thorium(IV); the relevant oxidation state and aqueous speciation, rather than the isotope label alone, govern chemical binding.

### 3.1. Radical and Radiation-Induced Grafting Strategies for Biodegradable Carbohydrate-Based Adsorbents in Radionuclide Removal

Among biodegradable materials, carbohydrate-based polymers such as cellulose, starch, and chitosan have attracted significant attention as adsorbent backbones. These polymers possess hydrophilic structures and contain reactive functional groups, including hydroxyl and amino moieties, which provide versatile platforms for further chemical functionalization.

Chitosan-based systems represent one of the most extensively studied carbohydrate adsorbents. Radiation-induced grafting has been successfully applied to chitosan matrices, enabling the incorporation of functional monomers and reinforcing nanostructures, leading to improved adsorption efficiency for radionuclides such as europium. For example, gamma radiation-induced grafting of acrylic acid and vinyl monomers onto chitosan combined with multiwalled carbon nanotubes resulted in composite materials with enhanced sorption performance and mechanical strength [80]. Similarly, hybrid composites incorporating Prussian blue analogs immobilized within chitosan-decorated carbon nanotubes have demonstrated high removal efficiency toward cesium and strontium ions, confirming the adaptability of carbohydrate matrices for complex radionuclide separation systems [81].

Cellulose and starch derivatives also exhibit strong potential as biodegradable adsorbent precursors. Graft copolymerization of acrylic acid and acrylamide onto carboxymethyl cellulose leads to superabsorbent materials with improved swelling properties and enhanced adsorption capability [79]. Likewise, graft copolymers based on starch modified with polyacrylic acid and polyvinylsulfonic acid have demonstrated effective adsorption of radionuclides such as cobalt and europium, highlighting the role of carbohydrate-based matrices in radionuclide remediation [82].

Despite their environmental advantages, native carbohydrate polymers exhibit several limitations that hinder their direct application as adsorbents. Their high solubility, excessive swelling, and relatively weak mechanical properties may lead to structural instability during adsorption processes. Furthermore, naturally occurring functional groups in carbohydrates often exhibit limited selectivity toward specific radionuclides, resulting in insufficient adsorption capacity. Therefore, chemical modification through grafting techniques becomes essential to enhance their performance.

Radiation-induced graft copolymerization (RIGC) represents a particularly effective and environmentally friendly approach for modifying carbohydrate polymers. This method enables uniform incorporation of functional monomers without the use of additional chemical initiators and allows precise control of graft density and polymer architecture [46]. The introduction of specific functional groups significantly improves adsorption selectivity and ion-exchange capacity. Carboxyl groups (-COOH/-COO^−^), typically introduced through acrylic acid monomers, provide strong cation-binding ability and facilitate pH-responsive swelling behavior, enhancing accessibility of adsorption sites. Sulfonate groups (-SO_3_^−^), introduced via vinylsulfonic acid, contribute strong acidic functionalities and improve ion-exchange efficiency, particularly in acidic radioactive environments. As negatively charged polyelectrolytes, sulfonated polymers enhance membrane selectivity and radionuclide binding capacity in challenging aqueous matrices. Additionally, specialized functional groups such as amidoxime have been widely recognized for their strong chelation ability toward uranium species, further demonstrating the importance of targeted grafting strategies in radionuclide adsorption materials [7].

Beyond improving adsorption selectivity, grafting also enhances the physical integrity of carbohydrate-based adsorbents. The formation of crosslinked or networked structures reduces polymer solubility and prevents disintegration during operation. Moreover, incorporation of reinforcing nanomaterials such as carbon nanotubes improves mechanical stability and increases available surface area for radionuclide binding [80]. These modifications ensure structural durability while maintaining the environmental advantages of biodegradable polymer backbones.

Carboxymethyl-cellulose graft copolymers are an established class of highly swelling superabsorbent networks [83]. El Saied et al. [79] prepared a magnetically recoverable superabsorbent nanocomposite, Na-CMC-g-P(AMPS-co-AA-co-AM)/Fe_3_O_4_ (SANCHs), by free-radical graft copolymerization of functional vinyl monomers onto carboxymethyl cellulose in the presence of Fe_3_O_4_ nanoparticles. Under optimized conditions (pH 9), the material exhibited preparation efficiencies of 78–82%, grafting yields of 1670–1790%, and gel contents of 76–80%. Its sulfonate, carboxylate, and amide groups supported electrostatic interactions and coordination with cationic species. Maximum adsorption capacities were 47.2 mg g^−1^ for ^60^Co, 43.0 mg g^−1^ for ^85^Sr, and 23.9 mg g^−1^ for ^134^Cs. High swelling facilitates uptake but can complicate handling and direct comparison with denser sorbents [79].

A chitosan–acrylic acid–1-vinyl-2-pyrrolidone/MWCNT composite exhibited adsorption capacities of 369.91 mg g^−1^ for ^60^Co, 456.46 mg g^−1^ for ^134^Cs, and 321.77 mg g^−1^ for ^152^/^154^Eu [80]. A distinct Prussian blue analog/chitosan/CNT composite adsorbed 205.1 mg g^−1^ of ^85^Sr and 219.8 mg g^−1^ of ^134^Cs [81]. These values illustrate the range achievable with different functional architectures, but they do not establish a universal ranking because the targets, feed compositions, pH values, contact times, and mass-transfer constraints differed between studies.

**Figure 6:**
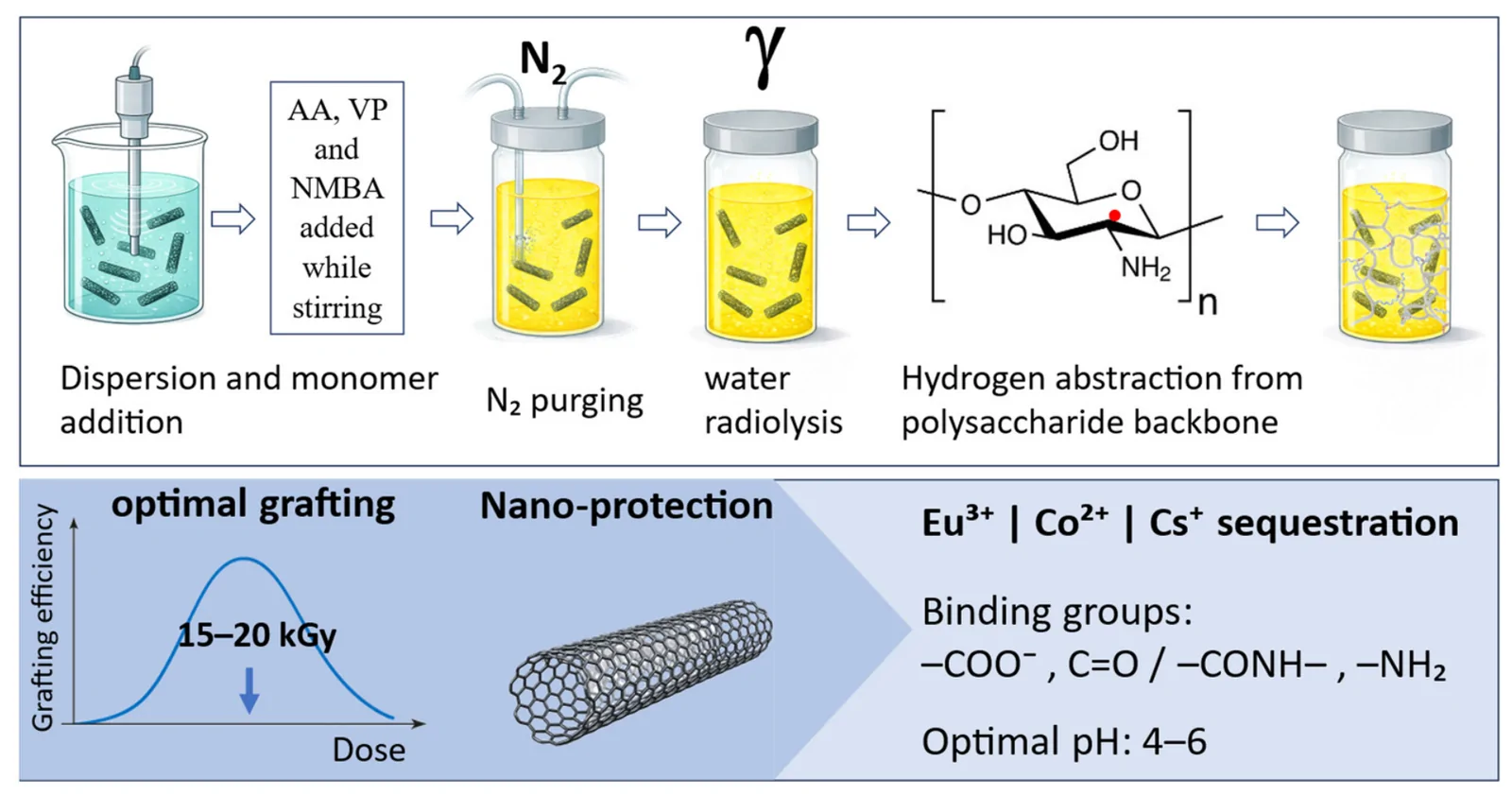
Author-created schematic of the γ-radiation-assisted preparation of CTS-g-AA/VP/o-MWCNT bionanocomposites and their radionuclide-binding mechanism, based on the experimental procedure reported in [80].

Abdelmonem et al. [80] prepared the chitosan–acrylic acid–1-vinyl-2-pyrrolidone/o-MWCNT composite by γ-radiation-induced graft copolymerization, using N,N′-methylenebis(acrylamide) (NMBA) as a crosslinker (Figure 6). Crosslinking prevents dissolution of the polymer network in water and regulates swelling, but excessive crosslink density can restrict monomer diffusion and reduce grafting efficiency. Increasing the o-MWCNT content slightly increased grafting efficiency and grafting yield by providing additional functional sites capable of radical initiation [80].

Grafting efficiency and grafting yield initially increased with absorbed dose because additional radical sites formed on the chitosan backbone. Above the optimum dose of approximately 15 kGy, both parameters declined as increasing network viscosity restricted monomer diffusion and chitosan degradation became more likely. The reported optimum formulation contained 30.0% comonomer, 1.0% chitosan, 0.25% o-MWCNTs, 0.2% NMBA, and an AA/VP ratio of 80:20 and was irradiated at 15 kGy. The final network structure reflects covalent bonding, dipole–dipole interactions, hydrogen bonding, and electrostatic forces. Consequently, monomer composition, absorbed dose, temperature, atmosphere, solvent, swelling, and monomer diffusion must be controlled together to optimize grafting and network homogeneity [80].

An increase in solution pH enhanced radionuclide adsorption up to mildly acidic conditions (pH 4–6), which represents the optimal binding window for the chitosan-based composite. At lower pH values, excessive H^+^ ions compete with metal cations for available binding sites, particularly carboxylate groups, thereby suppressing adsorption. As the pH rises toward 4–6, progressive deprotonation of functional groups (–COO^−^) increases the negative surface charge and strengthens electrostatic attraction and coordination with cationic radionuclides.

However, further increases in pH beyond this range do not improve adsorption and may lead to slight decreases in uptake. For multivalent ions such as Eu^3+^ and Co^2+^, higher pH can promote hydrolysis or partial precipitation, while increased OH^−^ concentration may also contribute to diffusion limitations within the polymer network. Consequently, the optimal adsorption performance is observed in the pH range of 4–6.

Under the optimum mildly acidic conditions, the chitosan composite adsorbed 321.77 mg g^−1^ of ^152^/^154^Eu, 456.46 mg g^−1^ of ^134^Cs, and 369.91 mg g^−1^ of ^60^Co [80]. These values should be interpreted within the reported pH, dose, composition, and contact-time conditions rather than as evidence that the material is universally superior to sorbents evaluated with other ions or feeds.

Li et al. [81] confined a Cu–Ni–Co hexacyanoferrate Prussian blue analog within a chitosan/CNT matrix. CNTs were oxidized with H_2_O_2_, and the composite was exposed to a 120 kGy γ dose to increase the number of active sites, surface area, and ion-uptake capacity. Transmission electron microscopy indicated a comparatively uniform composite. Sorption of Sr^2+^ and Cs^+^ was described by pseudo-second-order kinetics, consistent with a dominant chemisorption contribution, while the Elovich analysis indicated a stronger dependence of Cs^+^ uptake on the initial adsorption state [81].

Sr^2+^ and Cs^+^ transport in the CNT/chitosan/PBA composite involved intraparticle and film diffusion, with film diffusion particularly important for Cs^+^. Maximum adsorption capacities were 205.1 mg g^−1^ for Sr^2+^ and 219.8 mg g^−1^ for Cs^+^. Competing ions strongly affected uptake: the reported interference order was Na^+^ < Mg^2+^ < Ca^2+^ < K^+^ for Cs^+^ sorption and Ca^2+^ < Na^+^ < K^+^ < Mg^2+^ for Sr^2+^ sorption. The results indicate that K^+^ and Mg^2+^, respectively, may hinder Cs^+^ and Sr^2+^ recovery and may require matrix-specific suppression or pretreatment strategies [81].

El-Sweify et al. [78] investigated the selective removal and mutual separation of Co(II) and Eu(III), which may coexist in liquid waste from nuclear-reactor decommissioning. Five PAAm/MWCNT nanocomposites were prepared using different γ-radiation-induced polymerization or grafting strategies with N,N′-methylenebis(acrylamide) as a crosslinker. The mixtures were irradiated at 20 kGy in air at ambient temperature, at approximately 1.2 kGy h^−1^. Batch experiments used 0.01 g of sorbent and 5.0 mL of solution containing both ^60^Co(II) and ^152^/^154^Eu(III), with 24 h equilibration at 25 ± 1 °C. Performance was expressed using the distribution coefficient calculated from radioactivity before and after sorbent contact [78].

The adsorption behavior was strongly dependent on solution acidity. In the weakly acidic pH range investigated, the $K_{d}$ values for both Co(II) and Eu(III) generally increased with increasing pH, which was attributed to the reduced competition of H^+^ ions with metal cations for active sites on the polymer/MWCNT surface. However, the relative Co/Eu adsorption was not governed only by ionic charge. Although free Eu^3+^ would be expected to show strong electrostatic affinity because of its higher charge density, Eu(III) can undergo partial hydrolysis in weakly acidic aqueous media, forming species such as EuOH^2+^ and possibly Eu(OH)^+^_2_. These hydrated or hydrolyzed species may have reduced accessibility or lower effective affinity toward the sorbent compared with Co^2+^, which explains why, in most cases, Co(II) exhibited higher $K_{d}$ values than Eu(III) over the examined pH range. The extent of this effect depended on the composition and surface structure of the individual PAAm/MWCNT nanocomposite. Overall, the grafted nanocomposites showed markedly higher adsorption performance than the corresponding straight polymers, demonstrating their potential for radionuclide removal and, under selected conditions, Co/Eu separation [78].

Ghamry and Abdelmonem [82] prepared a biodegradable starch-g-poly(acrylic acid)-poly(vinyl sulfonic acid) network by γ-irradiation at 20 kGy, using N,N′-methylenebis(acrylamide) as a crosslinker. Acrylic acid introduced pH-responsive carboxyl groups, whereas vinyl sulfonic acid increased the density of anionic sulfonate binding sites. Uptake of Eu^3+^ and Co^2+^ was strongly pH-dependent and reached a maximum at pH 4–6. At lower pH, competition from H^+^ suppressed carboxylate binding; the higher Eu^3+^ affinity was attributed to its greater charge density and stronger interaction with oxygen-donor groups. Kinetic analysis indicated contributions from both intraparticle diffusion and surface-controlled chemisorption [82].

**Figure 7:**
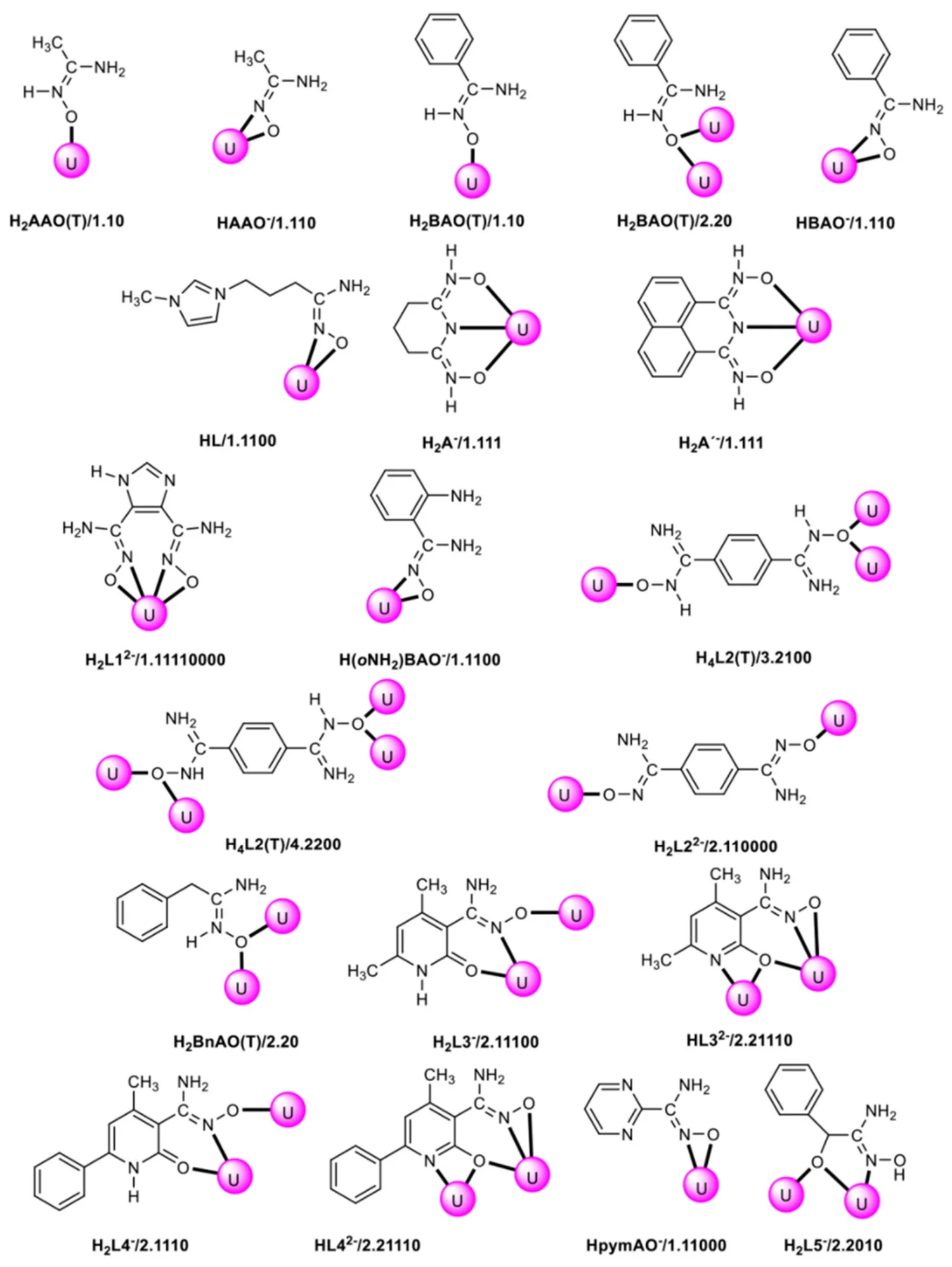
Coordination modes of amidoxime- and amidoximate-based ligands in structurally characterized uranyl(VI) complexes, described using Harris notation. The axial trans-oxo groups of the uranyl ion are omitted for clarity. Bold lines indicate coordination bonds. Reprinted from [84] under the terms of the Creative Commons Attribution 4.0 International License.

Uranyl ions can be recovered at very low concentrations using graft-functionalized polymers [7]. Seawater contains approximately 3 μg L^−1^ uranium, corresponding to a very large total resource because of the volume of the oceans. Amidoxime groups are widely investigated for this purpose because their amino nitrogen, oxime nitrogen, and oxime oxygen atoms can coordinate uranyl(VI), while their amphoteric character supports binding over a broad pH range, Figure 7 [84].

Acrylonitrile is commonly radiation-grafted onto polyethylene or polypropylene fabrics and subsequently converted to amidoxime groups by reaction of the nitrile functionality with hydroxylamine. Amidoxime adsorbents show affinity for uranyl(VI), which occurs predominantly as carbonate complexes such as [UO_2_(CO_3_)_3_]^4−^ in seawater [2]. Polyethylene-coated polypropylene nonwoven fibers provide chemically stable and mechanically useful substrates for this process. Copolymerization of acrylonitrile with hydrophilic monomers such as 2-hydroxyethyl methacrylate or methacrylic acid can further improve wettability [1,2]. Although performance has improved substantially, economical large-scale uranium recovery from natural seawater has not yet been demonstrated.

Fibrous amidoxime adsorbents were developed from radiation-grafted acrylonitrile on polyethylene fibers and then converted with hydroxylamine. Early marine tests demonstrated practical handling but also revealed limited durability associated with mechanical deterioration after amidoximation and non-uniform fiber assemblies [1]. Related PE/PP nonwoven systems have been evaluated at laboratory and pilot scales [8]. In one route, glycidyl methacrylate was radiation-grafted onto PE/PP, reacted with 3,3′-iminodipropionitrile, and converted with hydroxylamine to yield approximately 2 mmol of amidoxime groups per gram and a sorption capacity of 250 mg UO_2_^2+^ g^−1^ [9]. Field tests of amidoxime-based adsorbents yielded approximately 3.2–3.9 g U kg^−1^ of adsorbent after about 56 days in natural seawater [85].

Nuclear medicine also requires efficient treatment of radioactive waste and recovery of high-purity radionuclides. Gizawy et al. [86] investigated the recovery of carrier-free ^47^Sc^3+^, a theranostic radionuclide with a half-life of approximately 3.35 days, from irradiated natural calcium targets. A poly(acrylamide-co-acrylic acid)/MWCNT composite was prepared by γ-radiation-induced polymerization in the presence of N,N′-methylenebis(acrylamide). In a column system, approximately 80.5% of ^47^Sc^3+^ was eluted with 1 M HNO_3_; the remaining activity was subsequently recovered under acidic conditions through H^+^ competition for binding sites. The study demonstrates the potential of MWCNT-containing polymer composites for radionuclide purification in nuclear-medicine production [86].

Technetium-99m (^99^ᵐTc) is a metastable nuclear isomer widely used in diagnostic nuclear medicine because of its approximately 6 h half-life and convenient availability from ^99^Mo/^99^ᵐTc generators [87]. Kamal et al. [88] prepared ion-exchange membranes by radiation-induced graft copolymerization of acrylonitrile and acrylic acid onto high-density polyethylene, low-density polyethylene, and polypropylene films for the separation of ^99^ᵐTc from radioactive liquid waste. Post-treatment for 16 h at 90 °C with KOH, hydroxylamine, or HCl converted nitrile groups into carboxylate, amidoxime, or amide functionalities. Grafting depended strongly on solvent composition: water favored acrylic-acid grafting, whereas a 50/50 wt% H_2_O/DMF mixture maximized grafting of the acrylonitrile/acrylic-acid comonomer system [88].

KOH-treated membranes showed higher ^99^ᵐTc uptake than hydroxylamine-treated membranes. The degree of grafting remained below 83% for HDPE films. In LDPE, however, increasing grafting beyond 155% produced a dense, highly crosslinked network that restricted ^99^ᵐTc diffusion and reduced sorption. KOH-treated grafted polypropylene membranes provided the highest sorption among the tested materials and reduced the residual ^99^ᵐTc waste activity to 83.8% of its initial value. This result also shows that greater grafting is not automatically beneficial when crosslinking imposes mass-transfer limitations [88].

Suhartini et al. [89] grafted acrylic acid onto TiO_2_-densified cellulose derived from rice straw by simultaneous γ irradiation under oxygen-free conditions. The formulation contained N,N′-methylenebis(acrylamide) as a crosslinker and Mohr’s salt as a homopolymerization inhibitor and was irradiated at 20 kGy. The resulting Cell–TiO_2_–g–poly(AA)-co-MBA network exhibited a grafting degree of 56.37% and an adsorption capacity of 36.2 mg g^−1^ for U(VI), together with reduced swelling and improved acid resistance [89]. Akter et al. [90] grafted acrylonitrile and sodium styrene sulfonate onto nonwoven polyethylene at 50 kGy and converted the nitrile groups to amidoxime functionality. Maximum adsorption capacities were 138.95 mg g^−1^ for Cr(VI) at pH 1.43 and 60 °C after 24 h and 97.86 mg g^−1^ for Co(II) at pH 8.05 and 70 °C after 6 h. The markedly different optimum conditions demonstrate the importance of ion speciation and prevent direct ranking without matched test conditions [90].

Table 2 presents representative case studies illustrating how substrate selection, grafting route, and introduced functional groups determine the target-ion affinity and reported performance of graft-functionalized sorbents. Because the studies addressed different ions and applications, the comparison focuses on material-design strategies, evaluation methods, and study-specific limitations rather than on establishing a universal performance ranking.

### 3.2. Ion-Imprinted Polymers

**Figure 8:**
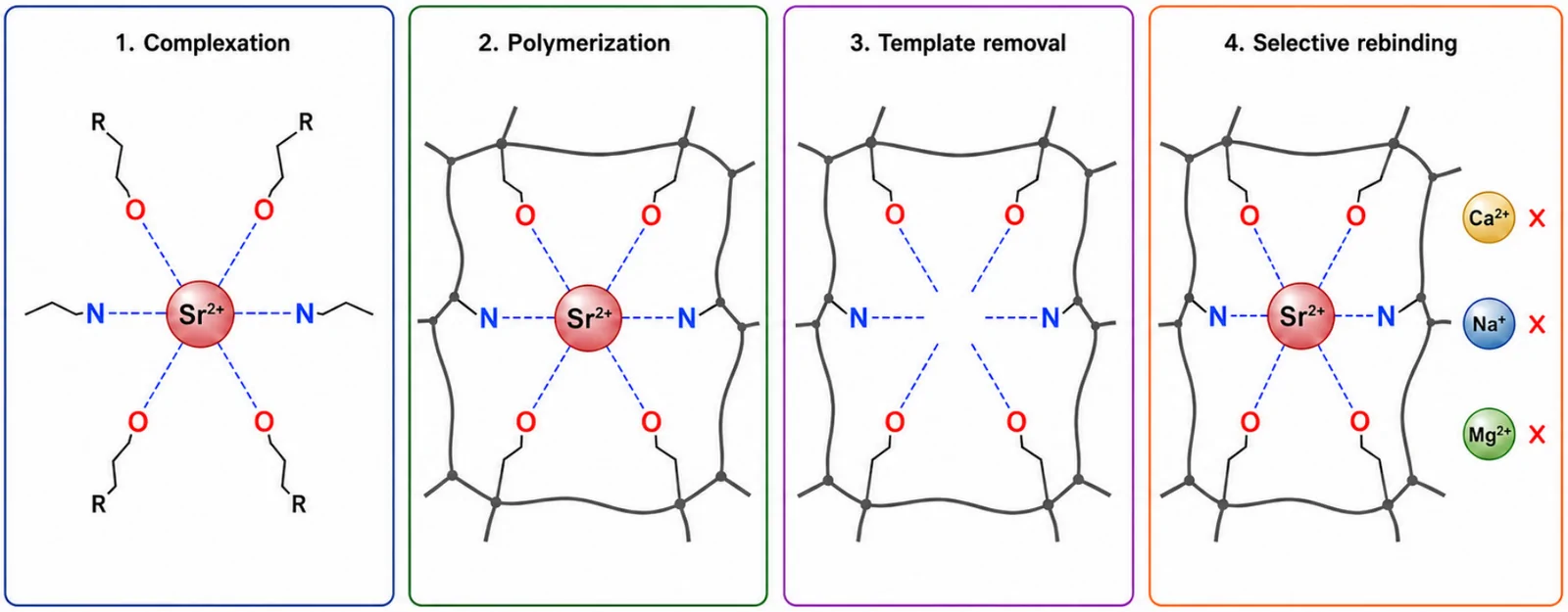
Schematic of the general concept of an ion-selective imprinted polymer, based on [92].

In another class of grafted materials, ion-imprinted polymers (IIPs, Figure 8) are commonly employed in radioactive adsorption and removal techniques. These crosslinked polymers contain specific cavities and binding sites complementary to the size and charge of target ions or molecules. In most cases, IIPs are made from a reaction composition that includes a crosslinker, a functional monomer, a template, and an initiator. Because of their selectivity and molecular recognition ability, IIPs have recently sparked a lot of interest in eliminating radionuclides such as Sr^2+^ and Co^2+^ ions [92]. The IIPs are resistant to temperature, pH, and pressure, offering greater physicochemical stability compared to biological recognition systems such as enzymes or antibodies.

IIPs are generally prepared through three main steps: (i) complexation of functional monomers with template ions, leading to self-assembly around the charged species; (ii) copolymerization in the presence of a crosslinker via thermal or photochemical initiation; and (iii) removal of the template ions to generate specific binding cavities complementary to the target ion. Because of their distinct binding sites for a specific ion’s size and charge, IIPs have extraordinary ion specificity. The adsorption performance of IIPs depends on several factors, including ionic charge, electronic configuration, oxidation state, ionic radius, and the coordinating ability of ligands present in the polymer matrix [92]. For instance, inorganic composites (such as SBA-15, palygorskite, and titanate whiskers) grafted on polyethyleneimine or chitosan and crosslinked with silane coupling agents or epoxy functionalized organic have been developed and proved to be efficient in the adsorption of Co^2+^ in Co-IIPs. Another instance is the use of chitosan and Potassium Titanate Whiskers (PTW) in the synthesis of IIP to selectively recover the Sr^2+^ from nuclear wastes. The PTW is chemically and mechanically stable, with exceptional wear resistance characteristics [92]. Preferential Sr^2+^-imprinted hybrid gels have also been synthesized using bis(trimethoxysilylpropyl)amine (TSPA), methacrylic acid (MAA), dicyclohexano-18-crown-6, and ethylene glycol dimethacrylate (EGDMA) as a crosslinker, providing high surface area and improved mechanical stability.

Thermoresponsive IIPs for Sr^2+^ extraction have been developed using SBA-15 mesoporous silica functionalized with magnetic polyethyleneimine. Free-radical polymerization of methacryloxypropyltrimethoxysilane, N,N′-methylenebisacrylamide (MBAA), and N-isopropylacrylamide (NIPAM) on the silica surface enabled formation of temperature-sensitive imprinted networks [92]. The resulting IIP-based material can then be used effectively in the separation/removal of the Sr^2+^ from various sources.

**Figure 9:**
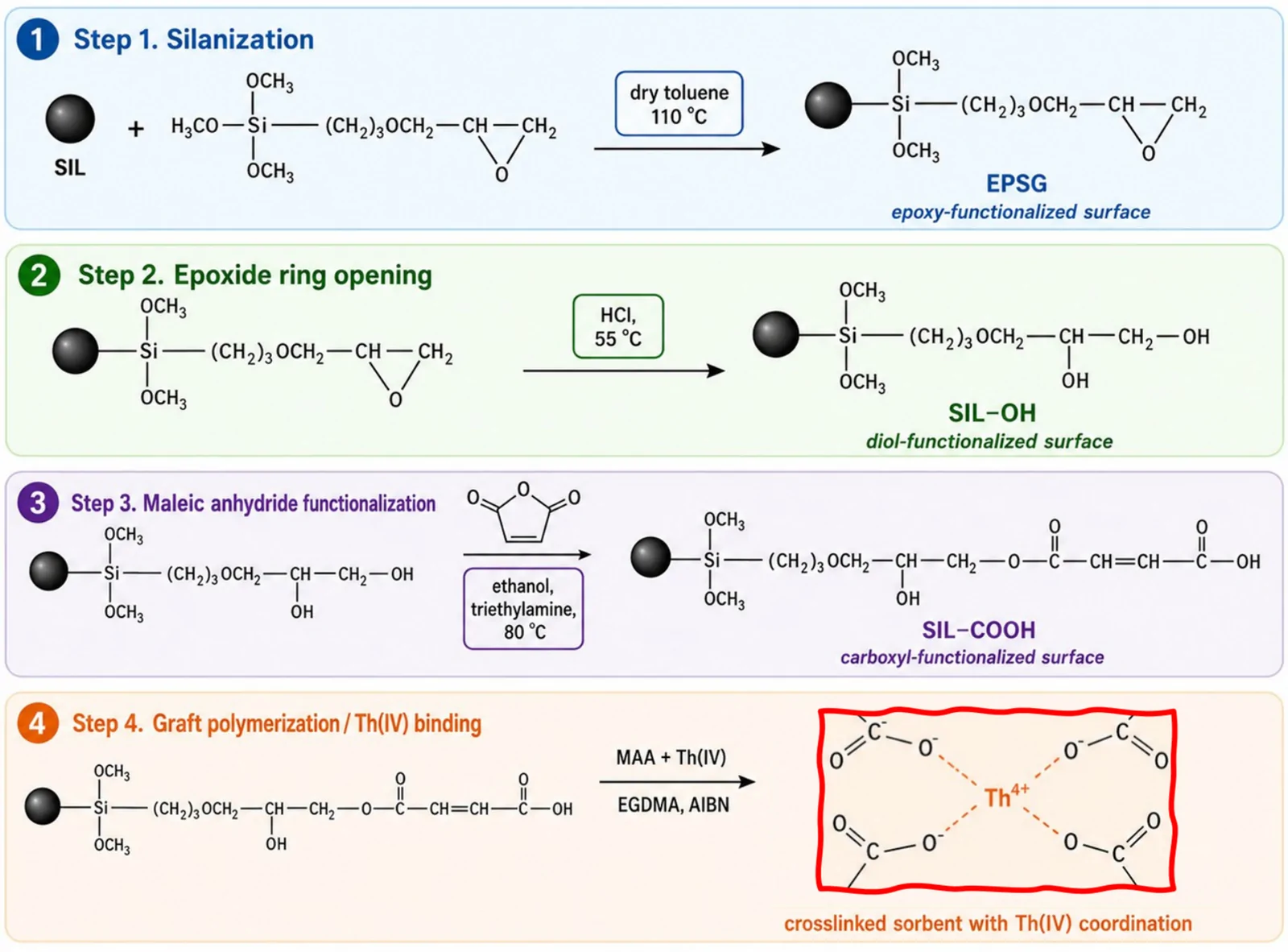
Schematic of the preparation and selective rebinding of Th(IV)-imprinted polymers, based on [91].

In one of the earlier examples [91], a Th^4+^-imprinted polymer was prepared on silica gel using a surface grafting approach with methacrylic acid (MAA) as the functional monomer. Th^4+^ ions were first complexed with MAA in methanol, followed by addition of the silica support (SIL-COOH), azobisisobutyronitrile (AIBN) as initiator, and ethylene glycol dimethacrylate (EGDMA) as crosslinker. After polymerization and subsequent removal of the template ions, specific binding cavities complementary to Th^4+^ were generated within the grafted polymer layer (Figure 9). The presence of Th^4+^ during polymer formation promoted an ordered arrangement of coordinating ligands. Upon template removal, well-defined binding sites remained, whereas non-imprinted polymers lacked comparable structural specificity. The sorption capacities of the imprinted and non-imprinted materials were 33.20 and 14.70 mg g^−1^, respectively [91]. The imprinted polymers for Th^4+^ depicted high selectivity, good reusability, high affinity, and a relatively fast kinetics process.

In summary, high equilibrium capacity alone does not establish a superior radionuclide sorbent. The most credible candidates combine selectivity in the presence of competing ions, rapid kinetics, mechanical stability, low secondary-waste generation, and reproducible regeneration over multiple cycles. Radiation dose must be optimized jointly with grafting yield and network density because excessive crosslinking can restrict diffusion, while chain scission can reduce mechanical lifetime.

## 4. Discussion and Future Directions

Radiation- and radical-induced graft copolymerization methods have found broad application in the fabrication of functional membranes for separation processes. In many industrial separation technologies, energy consumption constitutes a significant fraction of operational costs, particularly in thermally driven processes such as distillation or solvent extraction. The development of advanced membrane materials through grafting techniques enables the replacement or supplementation of such energy-intensive operations with membrane-based alternatives, contributing to reduced energy demand and potentially lower overall process expenditures [1].

The future of radiation- and radical-induced graft copolymerization will be shaped by process advantages that distinguish radiation-based methods from conventional chemical initiation. Radiation-induced grafting can proceed without added chemical initiators and may be conducted in aqueous or solvent-reduced systems. By controlling radiation type, absorbed dose, atmosphere, monomer diffusion, and reaction conditions, functionality can be introduced throughout a polymer or concentrated near its surface while preserving the bulk properties required for membrane and sorbent operation [1,2]. Detailed practical treatments of radiation-induced grafting further emphasize the need to balance radical generation, diffusion, grafting, crosslinking, and chain scission [93].

A further major strength of radiation techniques lies in their versatility in controlling the spatial extent of modification. Owing to the high penetration depth of ionizing radiation, reactive species can be generated throughout the bulk of a polymer matrix, enabling uniform volumetric functionalization when required. At the same time, through appropriate selection of irradiation conditions, monomer diffusion, and reaction parameters, grafting can be preferentially directed toward the near-surface region, preserving the physicochemical and mechanical integrity of the underlying material [1,2].

The effectiveness of radiation grafting is governed by the intrinsic radiation resistance of the polymer matrix. Material selection should prioritize backbone structures that favor radical stability and controlled crosslinking (e.g., polyethylene) or benefit from resonance stabilization (aromatic polymers such as polystyrene), while avoiding matrices prone to dominant chain scission, such as polypropylene with tertiary carbons or highly sensitive fluoropolymers like PTFE. The degree of crystallinity must also be carefully considered. Future material design should therefore focus on balancing radiation stability, controlled radical chemistry, and structural accessibility [38].

In direct connection with matrix selection considerations discussed above, carbohydrate- and polysaccharide-based materials represent a technologically attractive yet more demanding class of substrates. However, unlike radiation-resistant polyolefins or aromatic polymers, complex polysaccharides are intrinsically sensitive to ionizing radiation. Nevertheless, the examples presented in this work demonstrate that such challenges can be mitigated through careful control of irradiation parameters, optimized dose selection, protective comonomer systems, and tailored reaction environments. Continued research is therefore essential to balance grafting efficiency with structural preservation when employing radiation-sensitive biopolymer matrices [94].

Over the past three decades, controlled and living radical polymerization techniques have fundamentally reshaped the conceptual framework of polymer synthesis [95]. Methods such as ATRP, pioneered by Matyjaszewski et al. [23], together with RAFT and NMP, have established molecular-level control as a central paradigm in macromolecular engineering. These approaches enable predictable molecular weights, narrow dispersities, preserved end-group functionality, and access to complex architectures. In this context, future research in radiation- and radical-induced graft copolymerization should move beyond treating classical radiation grafting and modern controlled radical polymerization as separate domains. Bridging these classical and emerging methodologies represents a natural and strategically important evolution of the field [47]. In particular, RAFT-mediated and related living radical strategies enable precise regulation of graft density, chain length, and molecular weight distribution, facilitating the rational design of separation layers with tunable transport and binding characteristics. The integration of radiation-induced initiation within CRP frameworks therefore constitutes a key direction for the development of next-generation membranes and sorbents that are not only highly functional, but also structurally programmable at the molecular level.

Recent studies illustrate this broader design space. Electron-beam-induced in situ polymerization has been used to reweave ultramicroporous membrane architectures for CO_2_ separation [77], whereas γ-induced grafting has produced TiO_2_-reinforced cellulose adsorbents for U(VI) uptake [89] and bifunctional PE-based adsorbents for Cr(VI) and Co(II) removal [90]. Together, these examples show a transition from simple functional-group introduction toward deliberate control of transport pathways, reinforced matrices, and multifunctional binding environments.

Grafting can transform hydrophobic polymer matrices into hydrophilic, functionally active separation materials by introducing polar groups such as carboxylate, sulfonate, amide, or amidoxime functionalities. When confined to the surface or near-surface region, these groups can tune wettability and binding while preserving the mechanical properties of the bulk substrate. This offers greater chemical definition than nonspecific surface etching or activation [39]. Durable antifouling and antibacterial functionalities are also relevant to long-term membrane operation, although their stability must be demonstrated under realistic service conditions [96,97].

When grafting processes are transferred into aqueous environments, an additional mechanistic dimension must be considered. In radiation-driven systems, water radiolysis constitutes the primary physicochemical event upon irradiation in aqueous media. The formation of reactive species such as hydroxyl radicals, hydrated electrons, and hydrogen atoms governs subsequent initiation and propagation steps. Thus, the presence of water is not merely a solvent effect but a fundamental contributor to radical generation and reaction pathways. Importantly, reaction regimes differ substantially between hydrophobic matrices processed in nonpolar environments and hydrophilic systems operating under aqueous swelling conditions. Diffusion behavior, radical lifetimes, and functional group accessibility are dictated by distinct physicochemical constraints in each case. Recognizing these differences is essential for rational process design and for achieving reproducible graft architectures in both hydrophobic and hydrophilic systems [98].

Within this broadened framework of materials engineering, composite-based strategies emerge as a powerful complementary direction [40]. The incorporation of nanostructured fillers (such as carbon nanotubes, mesoporous silica, or titanate-based supports) can significantly enhance mechanical strength, introduce hierarchical porosity, and improve mass-transfer characteristics. Beyond reinforcement, such fillers may impart additional functional benefits, including adsorption activity or modified transport pathways. In radiation-driven systems, nanofillers can also influence radical distribution and potentially moderate radical-induced degradation in the surrounding polymer phase, contributing to improved structural stability [10].

Equally critical is the interfacial region between polymer matrix and filler phase. Processes occurring at phase boundaries differ fundamentally from those within the individual components, governing adhesion, stress transfer, radical localization, and functional group distribution. The interphase can therefore determine overall composite performance to a degree that exceeds the contributions of the isolated phases [99]. Future research should place stronger emphasis on interfacial engineering, compatibilization strategies, and controlled dispersion techniques to produce composite architectures with predictable structure–property relationships and long-term operational stability [100].

Industrial feasibility also depends on radiation infrastructure and quality assurance. Gamma facilities offer high penetration and batch treatment of dense or packaged substrates but require licensed sources, shielding, source security, and management of source decay. Electron-beam systems avoid sealed radioisotope sources and provide high dose rates and rapid on/off control, but penetration depth and line-of-sight geometry can limit product thickness. For both routes, scale-up requires validated dosimetry, dose-uniformity mapping, oxygen and temperature control, monomer recovery, ventilation, worker protection, and reproducible post-grafting conversion and washing. Economic assessment should therefore compare not only initiator and solvent savings but also facility utilization, throughput, energy demand, regulatory compliance, waste treatment, and the lifetime gained through regeneration [2,3,39,40].

Beyond the challenges discussed above, a more ambitious and still insufficiently explored direction concerns the transition from highly selective separation materials toward bio-inspired, functionally programmable platforms that combine molecular recognition with catalytic activity and large-scale process relevance [101]. In particular, ion- and molecularly imprinted systems demonstrate enzyme-like selectivity while retaining resistance to temperature, pH, and chemically aggressive environments, suggesting that radiation- and radical-grafted matrices could evolve from passive adsorbents into robust synthetic analogues of biological receptors [92]. Expanding this concept toward catalytically active, high-surface-area architectures remains largely underdeveloped within radiation-grafting research. Such architectures would be capable of selective activation, transformation, or degradation of target species under environmentally relevant conditions [102].

In parallel, the long-standing effort to recover uranium from seawater represents more than a resource-oriented objective; it constitutes a critical technological benchmark for the entire field. Despite decades of progress, a fully mature, economically viable large-scale solution has yet to be realized [102]. Achieving durable, regenerable, and kinetically efficient uranium-selective systems under real marine conditions would mark the first true demonstration of industrial-scale success for radiation-grafted functional materials. Such a breakthrough would not only validate the technology for nuclear resource recovery but also establish a scalable framework applicable to broader environmental remediation challenges, including selective extraction of critical elements, advanced water purification, and circular-economy processes. Developing such materials, particularly through integration with composite strategies and interphase engineering, could open new avenues in green catalysis, carbon capture chemistry, hydrogen-related processes, and environmentally adaptive separation technologies.

Commercialization should be evaluated through application-specific techno-economic analysis and life-cycle assessment. Relevant functional units include cubic metres of treated water, kilograms of removed contaminant, or membrane area operated over a defined lifetime. Such analyses should include polymer and monomer sourcing, irradiation energy and facility burden, solvent and wash-water recovery, module fabrication, regeneration chemicals, capacity loss, disposal of radioactive or metal-laden sorbents, and end-of-life options. Demonstrating stable performance in pilot-scale modules and real feeds is more informative than maximizing laboratory grafting yield or single-cycle adsorption capacity.

## 5. Conclusions

This narrative review examined radiation- and radical-induced graft copolymerization for environmental remediation and separation technologies, with emphasis on membrane-based gas and liquid separations, water purification, radionuclide removal, and resource recovery. Across these applications, grafting provides a means of localizing functional groups on or within a mechanically useful substrate, thereby tuning selective transport, adsorption, ion exchange, wettability, and fouling behavior.

The evidence also shows that performance is strongly conditional. Substrate chemistry, radical-generation route, monomer diffusion, grafting degree, crosslink density, radiation dose, atmosphere, solution composition, and target speciation jointly determine the outcome. High laboratory rejection or adsorption capacity should therefore not be treated as a universal material ranking when studies use different targets and test conditions. Membrane separation may reduce energy demand relative to thermally regenerated solvent processes, but radiation grafting itself is not intrinsically energy-saving; its feasibility depends on facility utilization, throughput, solvent and wash-water recovery, regeneration, service lifetime, and regulatory requirements.

The reviewed examples further distinguish three complementary functionalization strategies. Covalent grafting anchors introduced functionality to a polymer backbone; composite formation incorporates a pre-existing functional phase; and in situ polymerization generates a second polymer within an existing nanostructure. These approaches are best compared by functional-component localization, mode of retention, structural effect, fabrication complexity, leaching or rearrangement risk, regeneration behavior, and long-term stability rather than by single performance number.

Overall, radiation- and radical-induced graft copolymerization is a versatile materials-engineering platform, but translation beyond proof-of-concept studies requires validated performance in realistic feeds, repeated regeneration cycles, long-term mechanical and chemical stability, reproducible dosimetry, and application-specific techno-economic and life-cycle assessment. Integrating controlled radical polymerization, interfacial engineering, composite design, and molecular recognition offers a credible route toward more selective and durable membranes and sorbents.

## Figures and Tables

**Table 1:** Mechanistic and practical comparison of grafting approaches discussed in this review.

Method	Control Mechanism	Key Strengths	Main Limitations	Best-Fit Applications
Conventional free-radical grafting	Thermal/redox initiation followed by uncontrolled radical propagation; no reversible controlling agent or dormant functionality	Broad monomer and solvent tolerance; simple equipment	Broad dispersity; homopolymer formation; limited architectural control	Low-cost surface or bulk functionalization
ATRP	Reversible activation/deactivation by a transition-metal/ligand complex, commonly Cu(I)/Cu(II), acting on dormant alkyl-halide chain ends (C–X)	Good control of chain length and graft density	Metal residues; oxygen sensitivity; catalyst removal	Precisely controlled membrane brushes and PVC grafts
NMP	Reversible capping and uncapping by a nitroxide mediator, e.g., TEMPO or SG1; dormant chains are present as alkoxyamines	Simple mediator system; no metal catalyst	Elevated temperature; narrower monomer scope	Thermally robust graft architectures
RAFT	Reversible addition–fragmentation chain transfer mediated by a thiocarbonylthio CTA, Z–C(=S)–S–R, such as a dithioester, trithiocarbonate, xanthate, or dithiocarbamate	Broad functional-group tolerance; preserved end groups; architectural control	CTA selection; possible sulfur-containing residues; often needs a radical source	Controlled grafts, block structures, and radiation-initiated systems
Radiation-induced grafting	Ionizing radiation directly generates radicals in the substrate and/or monomer; no intrinsic controlling agent; simultaneous or pre-irradiation routes, optionally combined with RAFT	Initiator-free radical generation; spatial/depth control; complex geometries	Specialized facilities; dose optimization; chain scission/crosslinking competition	Membranes, fibers, resins, and radionuclide sorbents

**Table 2:** Representative graft-copolymer sorbents for radionuclide and metal-ion removal.

Substrate	Grafting Route	Target Ion(s)	Performance	Limitations
CMC/Fe_3_O_4_ hydrogel	Free-radical graft copolymerization and crosslinking. Sulfonate-, carboxyl-, and amide-containing grafted chains	Co(II), Sr(II), Cs(I)	Adsorption capacities of 47.2, 43.0, and 23.9 mg g^−1^ for Co(II), Sr(II), and Cs(I), respectively; optimum pH 9	High swelling; adsorption strongly dependent on pH and feed composition [79]
Chitosan–AA–VP/MWCNT	Gamma-radiation-induced graft copolymerization. Acrylic acid and 1-vinyl-2-pyrrolidone; carboxyl and amide groups	Co(II), Cs(I), Eu(III)	Adsorption capacities of 369.91, 456.46, and 321.77 mg g^−1^ for Co(II), Cs(I), and Eu(III), respectively; optimum pH 4–6	Excessive irradiation can cause chitosan-chain degradation and increase diffusion limitations [80]
Cellulose–TiO_2_–g–PAA	Simultaneous gamma-radiation-induced graft copolymerization; 20 kGy. Acrylic acid/poly(acrylic acid); carboxyl groups; MBA crosslinker.	U(VI)	Adsorption capacity of 36.2 mg g^−1^ for U(VI); grafting degree of 56.37%; synthesis performed under oxygen-free conditions	Moderate adsorption capacity; grafting and crosslinking must be balanced against swelling and acid resistance [89]
PE–g–AN/SSS after amidoximation	Simultaneous gamma-radiation-induced graft copolymerization; 50 kGy. Acrylonitrile and sodium styrene sulfonate; amidoxime and sulfonate groups after amidoximation.	Cr(VI), Co(II)	Adsorption capacities of 138.95 and 97.86 mg g^−1^ for Cr(VI) and Co(II), respectively; optimum conditions were pH 1.43, 60 °C, and 24 h for Cr(VI), and pH 8.05, 70 °C, and 6 h for Co(II)	Relatively severe and substantially different operating conditions for the two target ions [90]
Silica-supported Th(IV)-IIP	Surface graft copolymerization and ion-imprinting. Methacrylic acid and EGDMA; carboxyl groups and Th(IV)-imprinted cavities.	Th(IV)	Adsorption capacities of 33.20 and 14.70 mg g^−1^ for Th(IV) on the imprinted and non-imprinted polymers, respectively	Strong crosslinking can restrict site accessibility and slow mass transfer; selectivity is template-specific [91]

Note: Adsorption capacity, expressed in mg g^−1^, denotes the mass of the target ion adsorbed per unit mass of sorbent under the reported experimental conditions. Target species are reported by element and oxidation state rather than isotope because, for the adsorption mechanisms considered here, binding is governed by ionic speciation; the cited studies used particular radionuclides as experimental contaminants or tracers. Grafting degree and irradiation dose characterize material preparation rather than adsorption performance. Because the studies addressed different target ions and employed different experimental conditions, the reported values describe application-specific performance and should not be used to establish a universal ranking of the sorbents.

## Data Availability

No new data were created or analyzed in this study. Data sharing is not applicable to this article.

## Abbreviations

The following abbreviations are used in this manuscript:

AA	Acrylic acid
AAm/PAAm	(Poly)acrylamide
AIBN	Azobisisobutyronitrile
AN	Acrylonitrile
AO	Amidoxime
ATRP	Atom transfer radical polymerization
CNT	Carbon nanotube
CRP	Controlled radical polymerization
CTA	Chain transfer agent
CTS	Chitosan
DP	Degree of polymerization
EB	Electron-beam
EGDMA	Ethylene glycol dimethacrylate
G%	Grafting yield
GE%	Grafting efficiency
GMA	Glycidyl methacrylate
HA	Hydroxylamine
IIP	Ion-imprinted polymer
Kd	Distribution coefficient
MAA	Methacrylic acid
MBA/NMBA	N,N′-methylenebis(acrylamide)
MMM	Mixed matrix membrane
MWCNT	Multiwalled carbon nanotube
NIPS	Non-solvent induced phase separation
NMP	Nitroxide-mediated polymerization
PAI	Polyamide-imide
PAN	Polyacrylonitrile
PBA	Prussian blue analog
PDMA	Poly(dimethylaminoethyl methacrylate)
PDMS	Polydimethylsiloxane
PE	Polyethylene
PEO	Poly(ethylene oxide)
PI	Polyimide
PIM	Polymer of intrinsic microporosity
POEM	Poly(oxyethylene methacrylate)
PP	Polypropylene
PRUM	Polyolefin reweaved ultra-micropore membrane
RAFT	Reversible addition–fragmentation chain transfer
RG/RIGC	Radiation-induced grafting/Radiation-induced graft copolymerization
SANCHs	Superabsorbent grafted composite based on carboxymethyl cellulose and nanomagnetite
TB	Tröger’s base
THF	Tetrahydrofuran
UV	Ultraviolet
VBB	Victoria blue B
VP	1-vinyl-2-pyrrolidone
VSA	Vinyl sulfonic acid
